# Supplementary material for: Enzyme-Mediated Exponential Glucose Release: A Model-Based Strategy for Continuous Defined Fed-Batch in Small-Scale Cultivations
Source: Bioengineering (Basel). 2024 Jan 24;11(2):107. doi: 10.3390/bioengineering11020107 (PMC10886149; doi:10.3390/bioengineering11020107)
Supplement: Supplementary file 1 [file bioengineering-11-00107-s001.zip › bioengineering-2775234-supplementary.pdf]

Article

Enzyme-mediated exponential glucose release: A model-based strategy for continuous defined fed-batch in small-scale cultivations

Annina Kemmer [1], Linda Cai [1], Stefan Born [1], M. Nicolas Cruz Bournazou [1], and Peter Neubauer [1,*]

[1] Technische Universität Berlin, Institute of Biotechnology, Chair of Bioprocess Engineering, Berlin, Germany
* Correspondence: peter.neubauer@tu-berlin.de

Supplementary Data

Experimental conditions for model calibration experiments

A detailed overview of the experimental conditions of the cell-free glucose release experiments is given in Table S1.

Table S1. Detailed overview on the experimental conditions of the 24 enzymatic release experiments. The initial concentration of dextrin $\boldsymbol{D_0}$, glucose $\boldsymbol{G_0}$ and enzyme $\boldsymbol{E_0}$ were varied. To some experiments, dextrin $\boldsymbol{D_{add}}$ and glucose $\boldsymbol{G_{add}}$ was added after ~ 6h.

Exp.	D_0 [g L^{-1}]	G_0 [g L^{-1}]	E_0 [U L^{-1}]	D_{add} [g L^{-1}]	G_{add} [g L^{-1}]
1	15	0	10	-	-
2	15	0	20	-	-
3	15	0	10	-	3.75
4	15	0	20	-	3.75
5	15	0	10	5.25	
6	15	0	20	5.25	
7	30	0	10	-	-
8	30	0	20	-	-
9	15	7.5	10	-	-
10	15	7.5	20	-	-
11	30	15	10	-	-
12	30	15	20	-	-
13	30	0	10	-	7.5
14	30	0	20	-	7.5
15	30	0	10	10.5	-
16	30	0	20	10.5	-
17	15	3.75	10	-	-
18	15	3.75	20	-	-
19	15	3.75	10	-	3.75
20	15	3.75	20	-	3.75
21	30	7.5	10	-	-
22	30	7.5	20	-	-
23	30	7.5	10	-	7.5
24	30	7.5	20	-	7.5

Calculation of dilution by bolus additions

The addition of liquid boluses leads to rapid dynamics, resulting in almost infinite rates of change. This poses a challenge for numerical methods due to badly conditioned matrices and high numerical errors. Consequently, the macro-kinetic model does not incorporate bolus additions. Instead, they are delineated through algebraic equations associated to the discrete system input. If a liquid change occurs at $t = t_k$, the simulation is stopped, and the states are adapted accordingly:

$$x^+ = \begin{bmatrix} P^+ \\ S^+ \\ W_S^+ \\ E^+ \\ V^+ \end{bmatrix} == \begin{bmatrix} \dfrac{V^- P^- + \Delta V_P S_{i,glc}}{V^- + \Delta V_{add}} \\ \dfrac{V^- S^- + \Delta V_S S_{i,dex}}{V^- + \Delta V_{add}} \\ \dfrac{V^- S^- W_S^- + \Delta V_S S_{i,dex} W_{S,0}}{V^- S^- \Delta V_S S_{i,dex}}, \quad for\ \Delta V_S > 0 \\ \dfrac{V^- E^- + \Delta V_E S_{i,enz}}{V^- + \Delta V_{add}} \\ V^- + \Delta V_{add} - \Delta V_{sub} \end{bmatrix} \quad \text{(S1)}$$

Here, the minus-symbol refers to the state before the adaptation $x^-(t_k)$, and the plus-symbol to the state after the adaptation $x^+(t_k)$. The added volumes of the product glucose P, substrate dextrin S and enzyme E are given by $\Delta V_{add}(t_k) = \Delta V_P(t_k) + \Delta V_S(t_k) + \Delta V_E(t_k)$ and the subtracted volumes are denoted as $\Delta V_{sub}(t_k)$. The addition of the time reference (t_k) for states and added as well as subtracted volumes is omitted to improve readability. See table S2 for explanation of parameters $W_{S,0}$ and constants $S_{i,glc}$, $S_{i,dex}$ and $S_{i,enz}$

Subsequently, the simulation is restarted with the updated initial states after the system input. Please refer to [1] for the mathematical description of the microbial growth model including the algebraic equations related to system inputs.

Enzymatic fed-batch experiments

Table S2 contains the constants which were used, and parameters which were obtained from parameter fitting of the enzymatic fed-batch experiments. The full code is given in: https://git.tu-berlin.de/bvt-htbd/public/kemmer_2022_enzymatic-feed.git.

Table S2. Overview on parameters and constants. Description of parameters and constants which were obtained in the model fitting enzymatic fed-batch experiments are given, including the origin of the value.

Parameter / Constant	Description	Unit	Value	Origin / source
***E. coli* growth model:**				
$q_{s,max}$	Maximum specific substrate uptake rate	$gg^{-1}h^{-1}$	1.342	Data fitting
q_m	Specific maintenance coefficient	$gg^{-1}h^{-1}$	0.0519	Data fitting
$q_{Ac,max}$	Maximum specific acetate consumption rate	$gg^{-1}h^{-1}$	1.554	Data fitting
$q_{Ap,max}$	Maximum specific acetate production rate	$gg^{-1}h^{-1}$	0.186	Data fitting
K_S	Affinity constant for substrate (glucose) consumption	gL^{-1}	0.001	Data fitting
K_{qS}	Monod-type saturation constant for intracellular acetate production, dependent on the intracellular substrate flux q_S	$gg^{-1}h^{-1}$	3.872	Data fitting
K_A	Affinity for acetate consumption	gL^{-1}	0.420	Data fitting
$K_{i,AS}$	Inhibition of acetate uptake by glucose	gL^{-1}	0.929	Data fitting
$Y_{AS,of}$	Yield of acetate on substrate (overflow metabolism)	gg^{-1}	0.667	Data fitting
$Y_{XS,em}$	Yield of biomass on substrate, exclusive maintenance	gg^{-1}	0.518	Data fitting
Y_{XA}	Yield of biomass on acetate, exclusive maintenance	gg^{-1}	0.356	Data fitting
Y_{OS}	Oxygen used per gram of glucose metabolized	gg^{-1}	0.6	Data fitting
Y_{OA}	Oxygen used per gram of acetate metabolized	gg^{-1}	1.067	Stoichiometric constant [2]
Y_{PS}	Yield of product on substrate	gg^{-1}	2.00	Data fitting
$d_{Sox,P}$	Distribution constant defining fraction of q_{Sox} going into product formation	$\%100^{-1}$	0.123	Data fitting
**Glucose release model:**				
K	Michaelis constant	$g\ L^{-1}$	0.001	[3–5]
k_S	Catalytical constant for the susceptible substrate	$g\ (U\ h)^{-1}$	0.134 (data set 1), 0.976 (data set 2 and 3)	Data fitting
k_R	Catalytical constant for the resistant substrate	$g\ (U\ h)^{-1}$	0.00212	Data fitting
$W_{S,0}$	Initial relative amount of susceptible substrate	$g\ g^{-1}$	0.464	Data fitting

**Constant**				
H	Henry constant	% L g^{-1}	14000	Physical constant
τ	Response time of the oxygen sensor	h	65	Experimental data
$k_L a$	Volumetric oxygen transfer coefficient	h^{-1}	250	Data fitting
DOT^*	Dissolved oxygen concentration at saturation (aeration with 5 Lmin^{-1} air)	%	103	Experimental data
T	Temperature	° C	30	Experimental data
r_{evap}	Evaporation rate	Lh^{-1}	0.0005	Experimental data
$S_{i,glc}$	Glucose concentration in the feed	gL^{-1}	377.3 (data set 2), 336.3 (data set 3)	Input concentration
$S_{i,enz}$	Enzyme concentration in the feed	UL^{-1}	3000	Input concentration
$S_{i,dex}$	Dextrin concentration in the feed	gL^{-1}	150 (data set 1), 100 (data set 2 and 3)	Input concentration
C_A	Carbon concentration in acetate	mol C g^{-1}	0.033	Chemical formula [2]
C_S	Carbon concentration in the substrate (glucose)	mol C g^{-1}	0.033	Chemical formula [2]
C_X	Carbon concentration in the biomass	mol C g^{-1}	0.041	Stoichiometric analysis [2]
C_P	Carbon concentration in the product	mol C g^{-1}	0.044	Stoichiometric analysis

References

1. Kemmer, A.; Fischer, N.; Wilms, T.; Cai, L.; Groß, S.; King, R.; Neubauer, P.; Cruz Bournazou, M.N. Nonlinear State Estimation as Tool for Online Monitoring and Adaptive Feed in High Throughput Cultivations. *Biotechnology and Bioengineering* **2023**, doi:10.1002/bit.28509.
2. Xu, B.; Jahic, M.; Enfors, S.-O. Modeling of Overflow Metabolism in Batch and Fed-Batch Cultures of *Escherichia Coli*. *Biotechnology progress* **1999**, *15*, 81–90, doi:10.1021/bp9801087.
3. Fujii, M.; Kawamura, Y. Synergistic Action of α-Amylase and Glucoamylase on Hydrolysis of Starch. *Biotechnology and Bioengineering* **1985**, *27*, 260–265, doi:10.1002/bit.260270308.
4. Hiromi, K.; Ohnishi, M.; Tanaka, A. Subsite Structure and Ligand Binding Mechanism of Glucoamylase. *Molecular and Cellular Biochemistry* **1983**, *51*, 79–95, doi:10.1007/BF00215589.
5. Polakovič, M.; Bryjak, J. Modelling of Potato Starch Saccharification by an *Aspergillus Niger* Glucoamylase. *Biochemical Engineering Journal* **2004**, *18*, 57–63, doi:10.1016/S1369-703X(03)00164-5.

Disclaimer/Publisher's Note: The statements, opinions and data contained in all publications are solely those of the individual author(s) and contributor(s) and not of MDPI and/or the editor(s). MDPI and/or the editor(s) disclaim responsibility for any injury to people or property resulting from any ideas, methods, instructions or products referred to in the content.
